# ‘Sex’ in the cancer cell

**DOI:** 10.18632/oncotarget.2355

**Published:** 2014-08-15

**Authors:** Inam J. Lafta, Helen E. Bryant, Alastair S. H. Goldman

**Affiliations:** Department of Molecular Biology and Biotechnology, The University of Sheffield, Sheffield, UK

The development of better tools for diagnosis and more accurate prognosis of cancer includes the search for biomarkers; molecules whose presence, absence or change in quantity or structure is associated with a particular tumour or prognosis/therapeutic outcome. While biomarkers need not be functionally relevant, if their expression influences cell transformation or cancer cell survival, then they could also provide new targets for therapeutic drugs.

In recent years attention has been applied to a group of proteins known as cancer testis antigens (CT antigens) [1]. These proteins are products of genes whose expression was originally thought normally to be confined to the testis, yet they are expressed in tumour cells. CT genes are bound to serve a wide array of roles in the testes, which have many highly differentiated cell types and, uniquely, the specialised role of bearing a germline with cells passing through meiosis.

Early on, autologous typing of patient antibodies and T-cells demonstrated CT gene expression in tumours, the first example being MAGEA1 in melanoma [1]. Later serological screening of cDNA expression libraries identified further CT antigens including the meiosis specific synaptonemal complex protein 1 (SCP1) in malignant gliomas, breast, renal cell, and ovarian cancer [1]. More recently high throughput technologies have gradually increased the number of apparent CT antigens/genes [1, 2]. CT gene expression has been used as a prognostic/stratification tool to identify aggressive metastasis prone lung cancer [3] and chemo-resistance [4]. But wider analysis of gene expression suggests that activity of many CT is not wholly confined to the testis. Some CT genes are expressed in the central nervous system, and others are expressed in a range of normal tissues (see [5]). So, the CT label has been unfortunately applied as misnomer to some antigens/genes, and the generic group is probably not suitable as biomarkers. But, is there a subset of so-called CT genes that really are normally testis specific?

Following the discovery that ~25% of Drosophila genes misregulated during malignant brain growth are required in the germ line [6], Feichtinger, *et al* reasoned that the germline subset of testis expressing genes might be more reliably silent in normal somatic cells [5]. They termed these *meiotic cancer testis* (meiCT) genes. The functional classification of meiCTs is cautious, as it results from examining gene expression in isolated mouse meiotic cells. Mammalian meiotic function was not demonstrated for all genes, and there could have been non-meiotic cell contamination. Indeed, they found some putative meiCT genes, which are meiosis specific in lower eukaryotes, to be expressed in somatic tissue. However, other meiotic genes such as RAD21L and SMC1-beta appeared tightly restricted to the testis. A wider bioinformatic screen of putative meiCT genes using EST data, backed up by RT-PCR experiments, provided a larger group of 17 genes that appear to be expressed only in testes and cancer cell lines or tumour tissue. A further 5 genes were also expressed in the CNS, meaning their corresponding proteins should not normally be circulating around the body, so they remain potential biomarkers. More recently the same group have published an extension their work in *Oncoscience* [7]. They used a bioinformatics pipline to identify more putative meiCT genes followed by RT-PCR validation on a range of normal tissues, cancer cell lines and tumour tissues [7]. This identified a further 19 meiCT genes likely to be expressed in more than one tumour type. In both studies, a meta-analysis of patient microarray sets delivered positive hits for meiCTs, including for some that originally gave RT-PCR negative results in limited tumour samples.

Two aspects of the results are especially exciting. Firstly, a large proportion of positive hits indicated meiCT gene expression in ovarian cancer, for which potential CT biomarkers have been identified before (see [7]). Similar meiCT gene expression between testes and ovaries might seem like an obvious result since both organs undergo meiosis. But functional activity of the tesovarian and testes germlines does not simply overlap. Spermatogenesis is restricted to the adult, while oogenesis begins in the foetal ovaries, and then completes one cell at a time, monthly, in the adult. So association of meiCT gene expression with ovarian cancer hints at the possibility that these genes become deregulated in cancerous ovaries, or possibly they contribute to tumourogenesis. In either case, MeiCT genes/antigens could make good biomarkers in ovarian cancer.

Secondly, the expression of some meiCT genes makes oncogenic sense. For example the ADAM2 protein has a disintegrin and metalloprotease domain and is associated with cell-cell interactions (see [7]). These are properties that might well correlate with the invasive and metastatic characteristics of cancer cells. This is highly speculative as the functional relevance of meiCTs is yet to be confirmed, but it is worth considering that there is a prospect of some meiCT antigens not only serving as biomarkers but also having oncogenic properties, which could become targets for drug therapy. So, with more work and some luck, we may yet find that ‘sex’ in the cancer cells provides us with new tools to fight cancer.
